# Editorial: Neuro-derived control for interactive technology on unmanned robot systems

**DOI:** 10.3389/fnbot.2024.1360021

**Published:** 2024-02-22

**Authors:** Jiehao Li, Chenguang Yang

**Affiliations:** ^1^College of Engineering, South China Agricultural University, Guangzhou, China; ^2^Bristol Robotics Laboratory, University of the West of England, Bristol, United Kingdom; ^3^Department of Computer Science, University of Liverpool, Liverpool, United Kingdom

**Keywords:** human-robot interaction, sensor fusion, neuro-derived control, interactive technology, unmanned system

Unmanned robot systems with high delivery and flexible maneuverability have recently attracted increasing research interest, especially in engineering applications (Yang et al., 2018; Li et al., 2020, 2021; Huang et al., 2023). However, the interactive technology to execute autonomous navigation, exploration, tracking and other complex tasks in uncertain environments is still a key issue that needs to be solved urgently. Fortunately, neuro-derived control is readily available with the rapid development of sensors, which brings an opportunity to reconstruct a reliable human-like framework to approximate human dynamics. Furthermore, the neural network is a powerful tool to reflect the behavior of the human brain, allowing computer programs to recognize patterns and solve common problems in the fields of AI, machine learning, and deep learning. Therefore, advanced neuro-derived control methods for interactive technology on mobile robot systems are inspiring and promising topics. In this Research Topic, four papers are concerned with neuro-derived control on unmanned robot systems.

In collaborative scenarios involving human-robot interactions within shared workspaces, pursuing enhanced performance encounters a significant challenge—ensuring human safety. This necessity limits the speed and torque of robot drives, preventing any potential harm to the human body. This constraint becomes particularly pronounced in complex tasks involving adaptable human behavior, emphasizing the crucial need to uphold safe working distances and optimize task coordination. While a common approach involves a reactive servo response to the current human pose, this method falls short in exploiting the anticipated behavior of the human, rendering it less effective in responding promptly to rapid human motions. To address this limitation and enable the earliest possible adaptation of the robot's behavior, Lyu et al. introduced a novel brain-computer interface (BCI). This BCI, designed to detect the focal point of the human's overt attention, serves as a predictive indicator for imminent actions. Notably, the direct projection of stimuli onto the workspace facilitates seamless integration into existing workflows. Furthermore, we showcase the utility of the signal-to-noise ratio in the brain response, demonstrating its application in adjusting the velocity of robot movements based on the human's vigilance or alertness level. Through a comprehensive analysis of this adaptive system in a physical robot experiment, our findings indicate that the proposed method can potentially enhance collaboration efficiency and safety margins.

In grappling with the challenges posed by environmental uncertainty, multiagent systems encounter a pivotal hurdle—scalability. In response, Wei et al. introduced an innovative cooperative model for multi-agent systems grounded in a graph attention network. The approach meticulously considers the intricate relationships between agents and the complexities of continuous action spaces. Leveraging graph convolution and recurrent neural networks, they delineate these relationships in a dual-pronged manner. Graph convolution defines inter-agent relationships, while recurrent neural networks elucidate continuous action spaces. The optimization and modeling of the multiagent system are achieved by encoding interaction weights among agents through the graph neural network and weights between continuous action spaces via the recurrent neural network. To gauge the effectiveness of our proposed model, they conduct experimental simulations within a 3D wargame engine. The scenario involves unmanned air vehicles (UAVs) acting as attackers and radar stations serving as defenders, with both sides capable of detecting each other. The outcomes of these simulations unveil the superior performance of the model, surpassing the current state-of-the-art methods across dimensions of scalability, robustness, and learning efficiency.

Furthermore, in the case of the estimation of involuntary components in human arm impedance, Börner et al. discuss the importance of considering human feedback behavior for stable and efficient coordination in physical human-robot interaction, particularly in unpredictable tasks. In situations where voluntary cognitive feedback is too slow, humans rely on involuntary intrinsic and reflexive feedback. The combined effects of these feedback mechanisms and inertial characteristics are captured in involuntary impedance components. The manuscript introduces a method for estimating these involuntary impedance components in multi-joint movements of the human arm. This method involves applying force perturbations to elicit feedback jerks, isolating them using a high-pass filter, and limiting the estimation interval to exclude voluntary cognitive feedback. The approach utilizes a dynamic regressor representation and a first-order Taylor series expansion to create a linear model of the involuntary impedance components. The inertial parameters' constant values are estimated in a static posture maintenance task and then used to estimate the remaining components in a dynamic movement task. The method's superior performance is demonstrated through validation with simulated data of a neuromechanical model of the human arm, showing better estimation results for moderate movement velocities and less sensitivity to movement variability. The passage also mentions the successful application of real data in an experiment with human participants, highlighting the method's practicality and validity.

When it comes to different ways of performing a task with robotic support contributing to improved motor skills acquisition in dynamic scenarios, it is useful to consider the motor variability during robotic assistance enhances motor learning of dynamic tasks. For example, Özen et al. present the limitations of haptic guidance in robot-assisted training and propose Model Predictive Controllers (MPCs) as potential alternatives to enhance motor learning. While haptic guidance may improve task performance, it can hinder effort, interfere with the perception of environmental dynamics, and limit motor variability crucial for learning. The study conducted with 40 participants focused on a dynamic task involving swinging a virtual pendulum to hit targets. Two MPCs are designed: the end-effector MPC applies optimal forces on the end-effector, promoting motor variability and minimizing assisting forces; the ball MPC further reduces assisting forces on the virtual pendulum ball. The study compares MPCs to a control group without assistance and a group with conventional haptic guidance. The end-effector MPC proves effective, increasing movement variability, not hindering pendulum swing variability, and enhancing learning of task dynamics. The ball MPC improves performance and motivation during training but limits motor variability and the sense of agency, thereby limiting learning. Overall, training with MPCs, especially the end-effector MPC, shows promise in enhancing motor learning for tasks with complex dynamics, suggesting potential benefits for robotic training in neurological patients.

In summary, the topic session “*Neuro-derived control for interactive technology on unmanned robot systems*” presents a compelling case for the integration of neuroscientific principles into the control mechanisms of unmanned robot systems. With a solid foundation, thorough methodology, and real-world examples, the papers significantly contribute to the evolving field of robotics. Addressing the areas above for improvement would further strengthen the paper's impact and relevance.

## Author contributions

JL: Conceptualization, Data curation, Funding acquisition, Writing—original draft, Writing—review & editing. CY: Supervision, Writing—original draft, Writing—review & editing.
